# Drying-Rewetting and Flooding Impact Denitrifier Activity Rather than Community Structure in a Moderately Acidic Fen

**DOI:** 10.3389/fmicb.2016.00727

**Published:** 2016-06-01

**Authors:** Katharina Palmer, Julia Köpp, Gerhard Gebauer, Marcus A. Horn

**Affiliations:** ^1^Department of Ecological Microbiology, University of BayreuthBayreuth, Germany; ^2^Water Resources and Environmental Engineering Research Group, University of OuluOulu, Finland; ^3^BayCEER—Laboratory of Isotope Biogeochemistry, University of BayreuthBayreuth, Germany

**Keywords:** water table manipulation, climate change, wetlands, greenhouse gases, structural (functional) genes

## Abstract

Wetlands represent sources or sinks of the greenhouse gas nitrous oxide (N_2_O). The acidic fen Schlöppnerbrunnen emits denitrification derived N_2_O and is also capable of N_2_O consumption. Global warming is predicted to cause more extreme weather events in future years, including prolonged drought periods as well as heavy rainfall events, which may result in flooding. Thus, the effects of prolonged drought and flooding events on the abundance, community composition, and activity of fen denitrifiers were investigated in manipulation experiments. The water table in the fen was experimentally lowered for 8 weeks in 2008 and raised for 5.5 months in 2009 on three treatment plots, while three plots were left untreated and served as controls. *In situ* N_2_O fluxes were rather unaffected by the drought treatment and were marginally increased by the flooding treatment. Samples were taken before and after treatment in both years. The structural gene markers *narG* and *nosZ* were used to assess possible changes in the nitrate reducer and denitrifier community in response to water table manipulations. Detected copy numbers of *narG* and *nosZ* were essentially unaffected by the experimental drought and flooding. Terminal restriction fragment length polymorphism (TRFLP) patterns of *narG* and *nosZ* were similar before and after experimental drought or experimental flooding, indicating a stable nitrate reducer and denitrifier community in the fen. However, certain TRFs of *narG* and *nosZ* transcripts responded to experimental drought or flooding. Nitrate-dependent Michaelis-Menten kinetics were assessed in anoxic microcosms with peat samples taken before and 6 months after the onset of experimental flooding. Maximal reaction velocities *v*_*max*_ were higher after than before flooding in samples from treament but not in those from control plots taken at the same time. The ratio of N_2_O to N_2_O + N_2_ was lower in soil from treatment plots after flooding than in soil from control plots, suggesting mitigation of N_2_O emissions by increased N_2_O-reduction rates after flooding. N_2_O was consumed to subatmospheric levels in all microcosms after flooding. The collective data indicate that water table manipulations had only minor effects on *in situ* N_2_O fluxes, denitrifier abundance, and denitrifier community composition of the acidic fen, while active subpopulations of denitrifiers changed in response to water table manipulations, suggesting functionally redundant subpopulations occupying distinct ecological niches in the fen.

## Introduction

Peatlands cover about 3% of the earth's surface, are particularly important in mid- and high-latitudes, and store significant amounts of carbon and nitrogen (Gorham, 1991). Peatlands are sources and potential sinks of greenhouse gases such as methane (CH_4_) and nitrous oxide (N_2_O) (Christensen et al., 2003; Goldberg et al., 2008; Kolb and Horn, 2012). N_2_O is a major ozone-depleting substance and has a 300x higher global warming potential than CO_2_, (Ravishankara et al., 2009). N_2_O emissions from peatland soils are controlled by microorganisms. In water saturated systems, N_2_O is almost exclusively produced by denitrification [i.e., the sequential reduction of nitrate and/ or nitrite via nitric oxide (NO) to N_2_O and N_2_; Zumft, 1997]. Nitrate or nitrite are used as terminal electron acceptors by denitrifiers, and are supplied to peatlands by aerial precipitation, surface runoff, groundwater inflow, or nitrification in oxic zones (Conrad, 1996; Mosier et al., 1998; Goldberg et al., 2010; Lohila et al., 2010; Palmer et al., 2010). The extent of the oxic zone, and thus the magnitude of the nitrification process as a substrate producer for denitrification, is largely dependent on the water table level in peatlands (Lipson et al., 2012). Even though many pristine peatlands are net sources of N_2_O, (water-saturated) peatlands can be temporary sinks for N_2_O when nitrate/nitrite availability is low (Goldberg et al., 2008; Lohila et al., 2010; Palmer et al., 2010; Marushchak et al., 2011; Kolb and Horn, 2012; Palmer and Horn, 2012, 2015).

Peatland ecosystems are thought to be severely affected by future climate change (Gorham, 1991; Gong et al., 2012). Climate change is associated with increasing mean annual temperatures and an increased frequency of extreme weather events like prolonged dry periods and heavy rainfalls (Hartmann et al., 2013), which have the potential to lower and raise the water tables in soils, respectively (Gong et al., 2012). Those changes in watertable height will likely affect greenhouse gas emissions from peatlands. The effect of water table fluctuations on N_2_O emissions from wetlands is variably affected e.g., by the amplitude, frequency and duration of the water table fluctuations (Mander et al., 2011), ranging from enhanced emissions after long-term drainage (e.g., for forestry or agriculture), moderate short-term drainage or rapid flooding of dried peat soil (Martikainen et al., 1993; Goldberg et al., 2010; Maljanen et al., 2010; Jørgensen and Elberling, 2012) to reduced N_2_O emissions after flooding of peat soil (McNicol and Silver, 2014). Highly fluctuating water tables and rapid switching between water table heights lead to higher cumulative N_2_O emissions than stable water tables in wetland soils (Dinsmore et al., 2009; Mander et al., 2011; Jørgensen and Elberling, 2012; McNicol and Silver, 2014). In the past, much more attention has been paid to the effect of long-term (e.g., in multi-year drainage or peat restoration) than of short-term changes (i.e., on the basis of several weeks or months) in water table height on N_2_O fluxes from peatlands. Even fewer studies have focused on the effects of short-term intensive water table fluctuations on the denitrifier communities involved in N_2_O turnover in peatland soils (Kim et al., 2008). Kim et al. (2008) found a decline in *nirS* abundance in response to short-term drought in soil cores of bog and fen, suggesting a decline in proteobacterial *nirS*-hosting denitrifiers. Diversity of *nirS* was stable. However, effects of water table manipulations on denitrifier communities *in situ* are unclear to date.

Most denitrifiers are facultative aerobes and thrive under oxic as well as under anoxic conditions (Shapleigh, 2013). Indeed, oxygen rather than nitrate is the preferred electron acceptor for many denitrifiers, suggesting that oxic conditions will not impair denitrifiers and their genetic potential. Thus, we hypothesize that short-term water table fluctuations will change the denitrification activity of peat denitrifiers rather than their community composition. Thus, the aims of the present study were (i) to assess the effect of raised water tables on dentrification potentials in a model peatland, (ii) to determine the effect of lowered and raised water tables on the community composition of denitrifers, (iii) to detect possible changes in the active denitrifer communities, and (iv) to try to link the obtained results to observed *in situ* N_2_O fluxes.

## Materials and methods

### Study site and experimental setup

The minerotrophic fen Schlöppnerbrunnen is located in the Lehstenbach catchment (Fichtelgebirge, Germany; N 50° 07′ 53″, E 11° 52′ 51″). Please refer to Palmer et al. (2010) for a more detailed description of the sampling site. Mean air temperature was 6.9 and 6.6°C, while annual precipitation was 957 and 972 mm in 2008 and 2009, respectively. Three treatment and three untreated control plots (size 7.2 × 5 m) were established on the site and water table manipulations were performed as described (Estop-Aragonés et al., 2012, 2013). In brief, treatment plots were subjected to experimental drought and flooding in the summers of 2008 and 2009, respectively. The height of the water table was measured continuously in treatment and control plots (Figure S1). Experimental drought was achieved by rain water exclusion and drainage ditches in the time period between June 10th and August 7th 2008. PlexiGlas® roofs allowing for light penetration and above ground air movement were temporarily installed during the drought period, thus minimizing potential side effects. After the experimental drought period, roofs were removed, drainage was stopped, and drought plots were rewetted with artificial rainwater (103 mm within 8 h). Experimental flooding was achieved by irrigating the treatment plots with water from the nearby creek “Lehstenbach.” Creek water was spread onto the treatment plots via perforated tubes at an average rate of 70m^3^ per day and plot in the time period between May 14th and October 30th 2009. Maximum temperatures of drought and flooding plots in 5 cm of depth were ~1°C higher and 1.5°C lower than in control plots at the same time, respectively, indicating a minor effect of water table manipulations on the peat temperature regime (Estop-Aragonés et al., 2012).

Samples for molecular analyses were collected in both years, while samples for microcosm studies were collected in 2009 only due to the need of minimizing destructive samplings in 2008. In 2008, soil was sampled for molecular analysis before drought (June 09th) and at the end of the drought phase (July 27th). In 2009, samples were taken before flooding (i.e., before the onset of irrigation; May 11th) and after flooding (i.e., after irrigation had been discontinued; November 16th). Soil samples were taken with a peat soil corer from depth 0 to 40 cm. Soil samples for molecular analyses were separated into four layers (0–10, 10–20, 20–30, 30–40 cm), frozen immediately in liquid nitrogen, and stored at −80°C until use. Soil for microcosm studies was separated into two layers (0–20 and 20–40 cm) and stored at 4°C for max. 24 h prior to microcosm studies. Potential anaerobic microbial activities were significantly higher in 0–20 than 20–40 cm depth, and dissolved oxygen in pore water was close to air saturation deeper than 30 cm of depth in drought plots during water level drawdown (Wüst et al., 2009; Palmer et al., 2010; Estop-Aragonés et al., 2012). Air filled pore space was greater than 12% (up to 50% at the end of the drought period) from 0 to 20 cm of depth in drought plots (Estop-Aragonés et al., 2012). After the rewetting of drought plots, dissolved oxygen decreased to lower than 20% air saturation in 0–10 cm depth and declined to ~0 with increasing depth (Estop-Aragonés et al., 2012). In control plots, oxygen penetration was significant until 20 cm of depth (Estop-Aragonés et al., 2012). Thus, the results of (note: not the samples before) molecular analyses from 0 to 10 plus 10 to 20 cm, and from 20 to 30 plus 30 to 40 cm were pooled.

### Water table manipulations and effects on biogeochemistry

During the period of experimental drought (June 10th 2008 to August 7th 2008), water table heights ranged from −71 to −12 cm and from −90 cm (i.e., 90 cm below peat surface) to −14 cm in control and treatment plots, respectively (Estop-Aragonés et al., 2012; Figure S1). Average water table heights were −26.8 and −62.1 cm in control and drought plots, respectively, i.e., the water table was on average 35.4 cm higher in control than in treatment plots. Air filled pore space in 5 cm depth approximated 30% in control plots and 50% in drought plots. Water oxygen saturation approximated 0–1 and 80% in 30 cm depth of control and drought plots, respectively. Such strong lowering of the water table (i) increased dissolved oxygen levels close to saturation in more than 30 cm depth and (ii) significantly decreased concentrations of dissolved inorganic carbon in drought treatment relative to control plots (Estop-Aragonés et al., 2013). Nitrate concentrations were 0.02-0.15 mM in the pore water during the experimental period (Estop-Aragonés et al., 2013).

During flooding (May 14th 2009 to October 30th 2009), water table heights ranged from −49 to 1.6 cm and from −18 to 4.9 cm in control and treatment plots, respectively (Estop-Aragonés et al., 2012; Figure S1). Average water table heights were −15.4 and −0.7 cm in control and treatment plots, respectively, i.e., the water table was on average 14.7 cm higher in treatment than in control plots. Flooding (i) decreased dissolved oxygen (near 0 μmol/l in the final flooding phase), dissolved inorganic carbon and nitrate concentrations, and (ii) increased nitrate dependent electron turnover, acetate, and hydrogen concentrations relative to control plots (Estop-Aragonés et al., 2013).

### Assessment of *In situ* N_2_O-fluxes

*In situ* N_2_O-fluxes were measured by the closed chamber technique from late May to early November 2008 and from mid-April to mid-October 2009. The measurements were conducted as described earlier (Goldberg et al., 2010). In brief, three collars (1.15 l volume) were installed on each plot, and N_2_O fluxes were measured in regular intervals (2–4 and 1–2 times per month in 2008 and 2009, respectively). For the measurements, chambers of 4 l volume were placed on top of the collars, and the N_2_O concentration in the chamber headspace was measured after 0, 8, 16, 24, and 32 min using a photoacoustic infrared gas analyzer (Multigas Monitor 1312, INNOVA, Denmark). N_2_O flux rates were calculated based on the linear increase or decrease in N_2_O concentration in the chamber headspace.

### Assessment of denitrification potentials in soil microcosms

Denitrification potentials of fen soil (0–20 and 20–40 cm) at its *in situ* pH were assessed in nitrate-supplemented anoxic microcosms as described earlier (Palmer et al., 2010). In brief, one volume of fen soil was diluted with three volumes of water in 125-ml infusion flasks, the flasks were sealed with butyl-rubber stoppers and the airspace was purged with argon to achieve anoxic conditions. The flasks were preincubated at 15°C for ~16 h to reduce intially present nitrate. After preincubation, NaNO_3_ was added to the flasks (0–100 μM nitrate). Flasks were incubated for up to 12 h in the dark at 15°C, and N_2_O headspace concentrations in each flask were quantified at three timepoints using a Hewlett-Packard 5980 series II gas chromatograph equipped with an electron capture detector, and a Porapak Q-80/100 (Supelco, Bellefonte, PA) column (length, 4 m; inner diameter, 3.2 mm) with Ar-CH4 (95:5) as the carrier gas. Acetylene inhibition was used to distinguish between N_2_O production and total denitrification and to estimate the ratio of N_2_O to (N_2_O + N_2_) as described earlier (Yoshinari and Knowles, 1976; Palmer et al., 2010). N_2_O production rates and apparent kinetic parameters [Michaelis-Menten constants (*K*_*M*_), maximum reaction velocitites (*v*_*max*_), *v*_*max*_*/K*_*M*_] were determined as described (Palmer et al., 2010).

### Extraction of nucleic acids and reverse transcription

Nucleic acids from all sampled soil layers taken in 2008 were extracted using a bead-beating protocol (Griffiths et al., 2000) followed by separation of DNA and RNA using the Qiagen RNA/DNA Mini Kit (QIAGEN GmbH, Hilden, Germany) according to the manufacturer's instructions. Nucleic acids from all sampled soil layers taken in 2009 were extracted using a similar bead-beating protocol with the exception that an additional aluminum precipitation was performed prior to bead beating to remove humic acids (Persoh et al., 2008; Palmer et al., 2012). Although cell lysis procedures that are regarded as critical for the DNA/RNA extraction bias were essentially identical, comparison of community structure between the two years might be biased. Identical DNA/RNA extraction procedures were applied within each year, thus allowing for a meaningful analysis of the effect of water table manipulations on microbial community structure. Reverse transcription of extracted RNA into cDNA was conducted using the SuperScript VILO cDNA Synthesis Kit (Invitrogen, Karlsruhe, Germany) according to the manufacturer's protocol.

### Quantitative PCR

Quantitative kinetic real-time PCRs (qPCRs) of *narG* [narG1960f (TAY GTS GGS CAR GAR AA)/narG2650r (TTY TCR TAC CAB GTB GC); Philippot et al., 2002], *nosZ* [nosZF (CGC TGT TCI TCG ACA GYC AG)/nosZR (ATG TGC AKI GCR TGG CAG AA); Rich et al., 2003], and bacterial 16S rRNA genes [Eub341F (CCT ACG GGA GGC AGC AG)/Eub534R (ATT ACC GCG GCT GCT GG); Muyzer et al., 1993] from DNA samples were performed in three technical replicates per plot, sampling time point and soil depth as described (Zaprasis et al., 2010; Palmer et al., 2012). *narG* and *nosZ* amplified from cDNA obtained from the same fen samples during triplicate qPCRs yielded multiple bands on agarose gels and multiple melting points during melting curve analyses. Thus, *narG* and *nosZ* expression was not assessed. However, the bands with the correct size were excised from agarose gels and used for TRFLP analyses.

Obtained gene copy numbers were corrected for inhibition by spiking environmental DNA extracts with standard DNA as described earlier (Zaprasis et al., 2010; Palmer et al., 2012). Obtained inhibition factors ranged from 0.2–1.0, 0.1–1.0, and 0.1–1.0 for *narG, nosZ*, and 16S rRNA genes, respectively. Copy numbers of *narG, nosZ*, and 16S rRNA genes were compared between treatments and time points by Tukey's HSD test in IBM SPSS 22 after testing for normal distribution in R.

### Terminal restriction fragment length polymorphism

Triplicate qPCR reactions of *narG* and *nosZ* amplified from DNA or cDNA were pooled and gel purified using the Montage Gel Extraction Kit (Millipore Corporation, Bedford, MA, USA) prior to TRFLP analysis. The purified PCR products were digested with Mung Bean nuclease (New England Biolabs, Frankfurt am Main, Germany) to remove single-stranded DNA and reduce the probability of pseudo-terminal restriction fragments (Egert and Friedrich, 2003). The digested DNA was purified using the Millipore Multiscreen 96-well**-**Filtration System (Millipore Corporation, Bedford, MA, USA). *narG* and *nosZ* PCR products were digested with the restriction enzymes *Cfo*I and *Fnu4*HI (New England Biolabs, Frankfurt am Main, Germany), respectively. Polyacrylamide gel electrophoresis was performed as described previously (Palmer et al., 2010). Terminal restriction fragment (TRF) tables (i.e., relative fluorescence of TRFs per sample) were imported into Qiime 1.9 (Caporaso et al., 2010). Statistical differences between years, nucleic acid type and treatment were tested using the Qiime script compare_categories.py with the Adonis, anosim, mrpp, and permanova algorithms (for further details consult qiime.org). Results of the individual tests were compared. As obtained *P*-value estimations calculated by the different algorithms and the derived conclusions were similar, only *P*-values derived from Adonis are reported. Operational taxonomic units (OTUs as indicated by TRFs) that were differentially expressed between treatments and/or time points were identified using the Qiime script differential_otus.py. *In silico* digests of *narG* and *nosZ* obtained in an earlier study (Palmer et al., 2010) were used to identify TRFs. However, not all TRFs clearly affiliated with a taxon or sequencing based OTU of Palmer et al. (2010). Such TRFs were not linked to phylogeny.

## Results

### Effect of watertable manipulations on fen N_2_O fluxes

**Figure 1:**
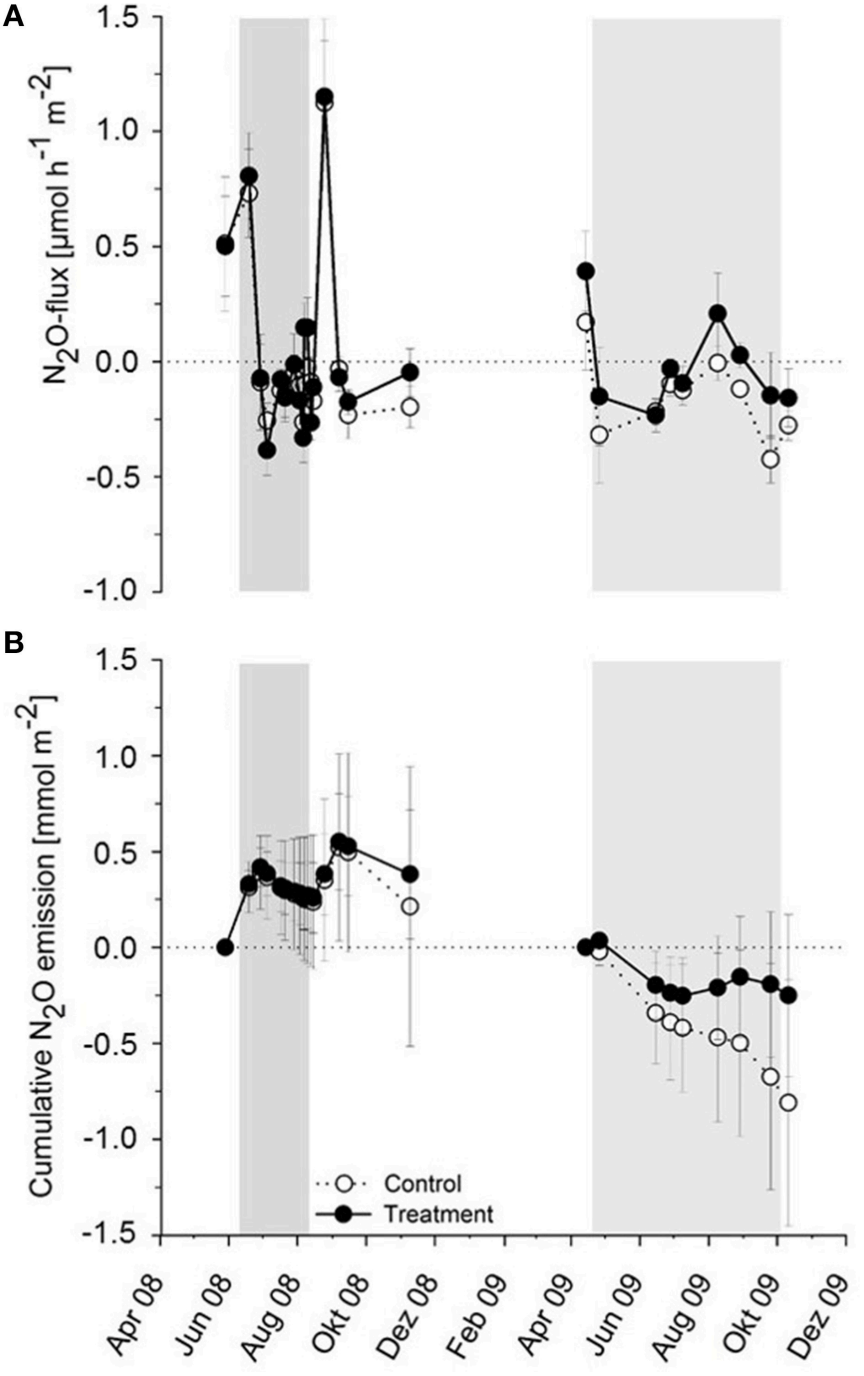
**Effect of experimental drought (2008) and prolonged flooding (2009) on (A) Mean and (B) cumulative N_2_O fluxes in Schlöppnerbrunnen fen**. Means of three plots per plot type (three measurements per plot) and standard errors are displayed. The period of the manipulation experiments is shaded in gray for each year.

N_2_O fluxes from Schlöppnerbrunnen fen were variable (Figure 1). In 2008 (drought experiment), mean N_2_O fluxes fluctuated between −0.4 μmol^*^h^−1^*^^m^−2^ (net N_2_O uptake) and 1.2 μmol^*^h^−1^*^^m^−2^ (net N_2_O emission). Differences between drought and control plots were marginal and were detected after the rewetting had occurred (Figure 1A). Cumulative N_2_O fluxes were positive in 2008, i.e., the fen was a net source of N_2_O in both drought and control plots (Figure 1B). In 2009 (flooding experiment), mean N_2_O fluxes ranged from −0.4 μmol^*^h^−1*^m^−2^ (net N_2_O uptake) to 0.4 μmol^*^h^−1^*m^−2^ (net N_2_O emission). Differences between treatment and control plots were marginal in both years, indicating that N_2_O fluxes were essentially unaffected by the experimental drying or flooding (Figure 1A). In 2009, cumulative N_2_O fluxes were negative in both plot types, indicating (i) net N_2_O uptake in both flooding and control plots and (ii) differences between the years 2008 and 2009 (Figure 1B).

### Effect of watertable manipulations on copy numbers of *narG, nosZ* and 16S rRNA genes

**Figure 2:**
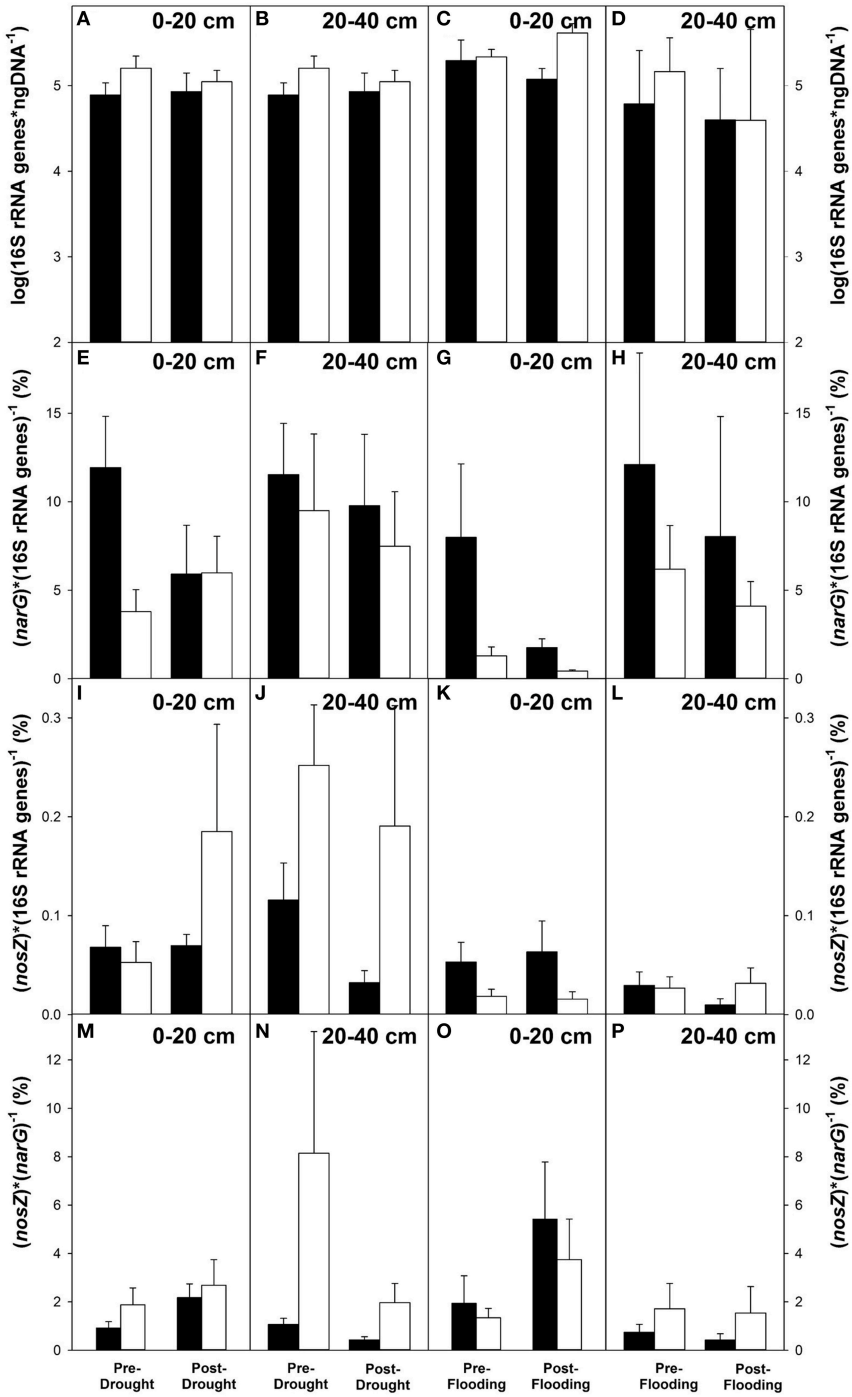
**Effect of experimental drought (left) and prolonged flooding (right) on 16S rRNA gene copy numbers (A–D), relative abundances of *narG* (E–H), and *nosZ* (I–L) as well as on the ratio of *nosZ* to *narG* (M–P) in 0–20 and 20–40 cm fen soil**. Black bars represent control plots, white bars represent treatment plots. Mean values from triplicate analyses of all plots of one plot type and depth 0–20 or 20–40 cm were calculated and are displayed with error bars. Pre-drought samples were taken on June 09th 2008, post-drought samples were taken on July 27th 2008. Pre-flooding samples were taken on May 11th 2009, post-flooding samples were taken on November 16th 2009.

Experimental drought successfully changed water levels and soil biogeochemistry relative to control plots as did experimental flooding (Estop-Aragonés et al., 2012; Figure S1; please refer to data from our colleagues presented in the Materials and Methods section for further details on the effect of water table manipulations on biogeochemistry). Inhibtion-corrected 16S rRNA gene copy numbers were averaged for depths of 0–20 and 20–40 cm, for different plot types, treatments, and time points. Averaged copy numbers ranged from (7.8 ± 3.0) × 10^4^ to (4.1 ± 1.2) × 10^5^ per ng DNA (Figures 2A–D). 16S rRNA gene copy numbers from both plot types and at all sampling time points were similar (*P* > 0.1). Inhibtion-corrected copy numbers of *narG* and *nosZ* amplified from extracted DNA ranged from 0.4 to 12% and from 0.01 to 0.25% of bacterial 16S rRNA gene copy numbers, respectively (Figures 2E–L). The following effects reflect tendencies rather than significant differences: In drought plots, relative abundances of *narG* and *nosZ* were similar (*P* = 0.9) and 3x higher (*P* = 0.3), respectively, after than before drought in 0–20 cm peat soil, while relative abundances of both genes were similar before and after drought in 20–40 cm soil (*narG*: *P* = 0.7, *nosZ: P* = 0.9; Figures 2E–F,I–K). When the same time points were compared, relative abundances of *narG* and *nosZ* from control plots were 2x lower (*P* = 0.4) and similar (*P* = 0.99), respectively, in 0–20 cm peat soil, and similar (*P* = 0.99) and 2.5x lower (*P* = 0.8), respectively, in 20–40 cm peat soil (Figures 2E,F,I,K, respectively).

Relative abundances of *narG* were marginally lower after than before flooding in treatment plots in both depths (*P* ≥ 0.9), while relative abundances in control plots were about 3x lower (*P* = 0.1) in 0–20 cm peat soil when the same time points were compared (Figures 2G,H). Relative abundances of *nosZ* were similar at the after and before flooding time points in treatment plots and control plots at the same time in 0–20 cm peat soil (*P* ≥ 0.9), and marginally lower after flooding of treatment plots in 20–40 cm peat soil from control plots (*P* = 0.7; Figures 2K,L). *narG* was on average 12x to 240x more abundant than *nosZ* (Figures 2M–P). The ratio of *nosZ* to *narG* was marginally higher in treatment than in control plots at both sampling time points during the drought experiment in 0–20 cm peat soil (*P* ≥ 0.7), and no effect of the drought treatment on *nosZ* to *narG* ratios was observed (Figure 2M). In 20–40 cm peat soil the ratio was higher before than after drought in treatment plots (*P* = 0.2; Figure 2N). During the flooding experiment, a minor increase in the ratio of *nosZ* to *narG* in treatment plots was observed after relative to before flooding in 0–20 cm (*P* = 0.7) but not in 20–40 cm peat soil (*P* = 0.99; Figures 2O,P).

### Effect of watertable manipulations on community composition of *narG* genes

**Figure 3:**
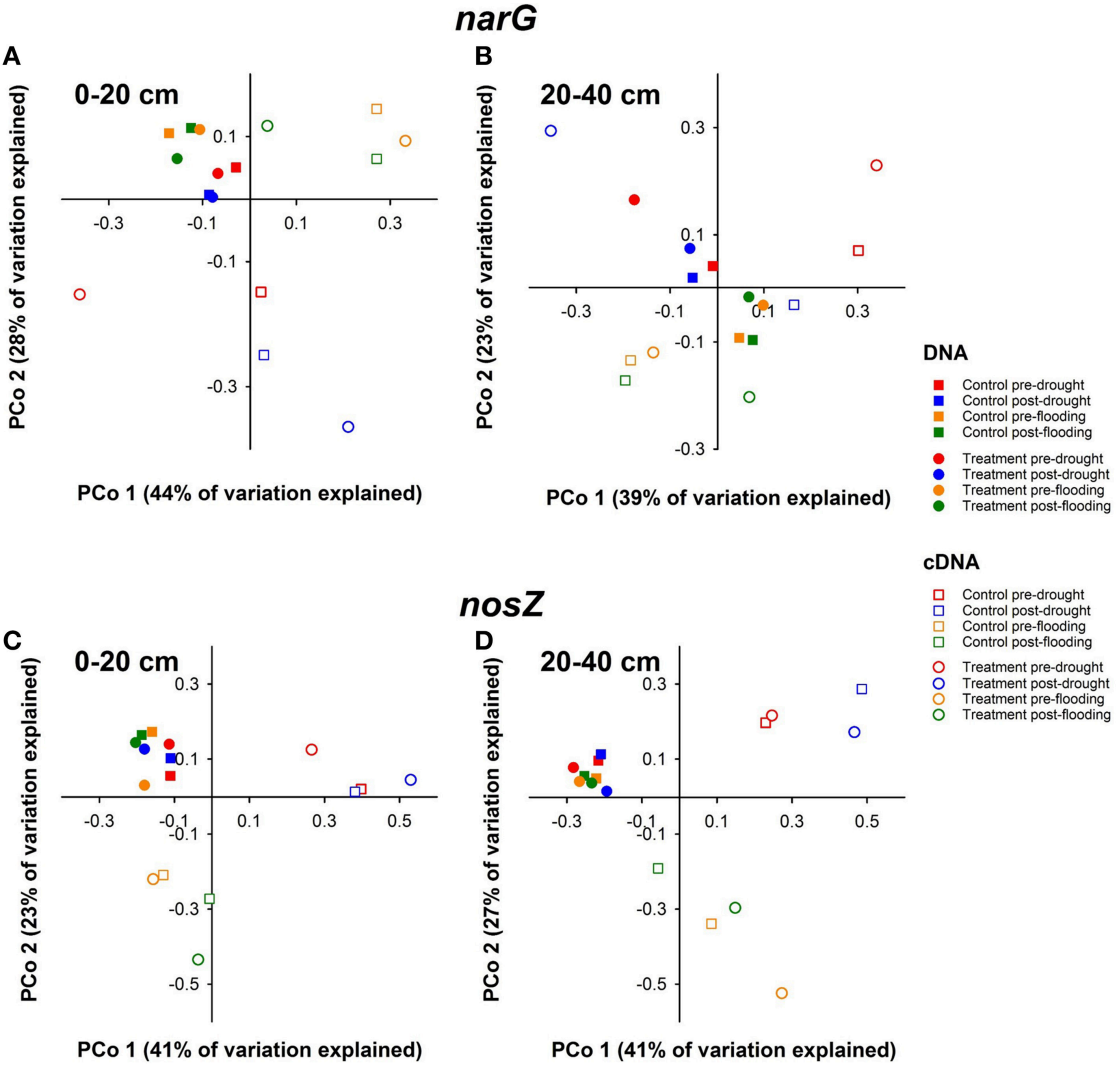
**Effect of artificial drought and prolonged flooding on the community composition of *narG* (A,B) and *nosZ* (C,D) in DNA and cDNA samples from fen soil from 0 to 20 cm (A,C) and 20 to 40 cm (B,D) depth**. Principal coordinate analysis was conducted in Qiime using the average relative abundances of TRFs per plottype and time point as input. Pre-drought samples were taken on June 09th 2008, post-drought samples were taken on July 27th 2008. Pre-flooding samples were taken on May 11th 2009, post-flooding samples were taken on November 16th 2009.

Principal Coordinate Analysis of *narG* TRFLP patterns obtained from DNA samples indicative of the genetic potential for dissimilatory nitrate reduction revealed that the detected *narG* community composition was similar at all time points, as DNA samples clustered closely together in the PCoA plot (Figures 3A,B). This was observed in both depths, even though the clustering was slightly more pronounced in 0–20 cm depth (Figure 3A). Up to 9 and 13 major TRFs of *narG* were detected in DNA samples from both depths in 2008 and 2009, respectively, and the relative abundances of the individual TRFs were similar in treatment and control plots at all sampling time points. Detected TRFs were indicative of uncultured soil and sediment organisms related to *Deinococcus-Thermus* sp. of FEN CLUSTER 7, *Actinobacteria*-related uncultured soil bacteria of FEN CLUSTER 6, and uncultured Proteobacteria of FEN CLUSTERs 1–5 (Palmer et al., 2010) (Figures S2A,B,E,F). No statistically significant differences were detected between the treatments and time points (*P* > 0.2).

### Effect of watertable manipulations on community composition of *narG* transcripts

Control plot samples obtained before and after treatments clustered more closely together than treatment plot samples based on TRFLP patterns of *narG* amplified from cDNA indicative of active nitrate reducers (Figures 3A,B). In 2008, when the drought experiment was performed, cDNA TRFLP patterns of *narG* differed already before drought in 0–20 and 20–40 cm peat soil (Figures 3A,B). The relative abundances of the two most abundant TRFs (23 and 57 bp) were similar in control plots in both soil layers (*P* > 0.9), while their relative abundances differed or tended to differ in treatment plots before and after drought (*P* = 0.01 and *P* = 0.35 for 23 and 57 bp TRF, respectively, in 0–20 cm soil; *P* = 0.04 for both 23 and 57 bp TRF in 20 to 40 cm soil; Figures S2C,D). This indicates that the activity of the groups behind those TRFs, i.e., *Deinococcus-Thermus* and *Actinobacteria* related uncultured fen nitrate reducers, is strongly affected by the experimental drought conditions (Figures S2C,D).

In 2009 when the flooding experiment was performed, cDNA TRFLP patterns of *narG* from control plots clustered together in the PCoA plots (Figures 3A,B). The samples from control and flooding plots taken before the flooding treatment also clustered together, indicating that the active communities were rather similar in control and treatment plots before the onset of flooding. The samples from treatment plots after treatment clustered separately in the PCoA plots, suggesting an effect of flooding on active *narG* expressing microbes (Figures 3A,B). Marginal differences in the relative abundance of TRFs indicative of *Deinococcus-Thermus* and *Actinobacteria* related uncultured fen nitrate reducers suggest that those groups responded to flooding in upper peat soil (Figure S2G).

### Effect of watertable manipulations on community composition of *nosZ* genes

TRFLP patterns of *nosZ* amplified from DNA were similar in 2008 (drought) and 2009 (flooding) and in both control and treatment plots (*P* > 0.2; Figures 3C,D). The five most prominent TRFs were indicative of uncultured soil organisms related to *Bradyrhizobiaceae* of FEN CLUSTER 1 and *Rhodospirillaceae* of FEN CLUSTERS 3–5 within the *Alphaproteobacteria* (Palmer et al., 2010) (Figures S3A,B,E,F).

### Effect of watertable manipulations on community composition of *nosZ* transcripts

TRFLP patterns of *nosZ* obtained from cDNA differed from TRFLP patterns obtained from DNA in both years (*P* = 0.001; Figures 3C,D). The TRFs that were most prominent in DNA samples accounted only for 22–52 and 38–90% of the TRFs in cDNA samples in 2008 and 2009, respectively. In 2008, samples from control plots were rather similar in 0 to 20 cm depth at both sampling timepoints, while there were differences between samples taken before and after drought from treatment plots (Figure 3C). In 20 to 40 cm depth, samples from treatment plots taken before and after drought and those of control plots taken at the same time clustered together, and samples taken after drought were different from the samples taken before drought as well as from each other (Figure 3D).

Significant differences between treatments and sampling time points were not detected (*P* > 0.12 in both soil layers) based on the overall TRFLP patterns obtained from cDNA samples in 2008 when the drought experiment was performed. Even though the overall community strucure was rather similar, 11 TRFs were expressed differentially in different plots or time points (Table 1; Figure S3). Those TRFs were indicative of uncultured *Bradyrhizobiaceae* and *Rhodospirillaceae* (Figure S3). Thus, data suggests that experimental drought affected activities of certain uncultured fen denitrifiers of the *Alphaproteobacteria*.

**Table 1:** **Important *nosZ* cDNA TRFs responding to the drought treatment**.

			**Drought treatment**		**Control**	
			**Pre-**	**Post-**	**Pre-**	**Post-**
Drought treatment	Pre-	0–20	X		80↑^*^	n.a.
					390↑^***^	
					700↓^***^	
		20–40	X	80↑^***^	103↓^***^	n.a.
				103↓^***^	149↑^***^	
				118↑^***^	165↑^**^	
				135↑^***^	419↑^**^	
				149↑^**^		
				165↑^***^		
				298↓^*^		
	Post-	0–20		X	n.a.	
		20–40	80↓^***^	X	n.a.	103↓^**^
			103↑^***^			118↓^***^
			118↓^***^			
			135↓^***^			
			149↓^**^			
			165↓^***^			
			298↑^*^			
Control	Pre-	0–20	80↓^*^	n.a.	X	80↓^*^
			390↓^***^			390↓^***^
			700↑^***^			700↑^***^
		20–40		n.a.	X	
	Post-	0–20	n.a.		80↑^*^	X
					390↑^***^	
					700↓^***^	
		20–40	n.a.	103↑^**^		X
				118↑^***^		

*Arrows indicate higher or lower relative abundances of TRFs when conditions are compared from left to right*.

* P < 0.10;

** P < 0.05;

*** *P < 0.01*.

*Empty fields indicate that no differentially expressed TRFs were detected in that comparison. n.a., not analyzed*.

In 2009, when the flooding experiment was performed, samples taken before flooding of treatment plots from control and treatment plots clustered together in the PCoA plots in 0–20 cm depth, while they scattered in 20–40 cm depth (Figures 3C,D). Statistically, the overall TRFLP patterns were rather similar in both treaments and at both sampling time points in the upper layer (*P* = 0.3), indicating a minor effect of flooding on the active denitrifier community in the upper peat soil. On the contrary, the overall TRFLP patterns in the lower peat soil differed significantly between the plottypes and time points (*P* = 0.02). Seven TRFs were expressed differentially in different plots or time points (Table 2; Figure S3). The data suggests that activities of uncultered *Bradyrhizobia*-like denitrifiers were impaired by experimental flooding.

**Table 2:** **Important *nosZ* cDNA TRFs responding to the flooding treatment**.

			**Flooding Treatment**		**Control**	
			**Pre-**	**Post-**	**Pre-**	**Post-**
Flooding treatment	Pre-	0–20	X	90↓^***^		n.a.
		20–40	X	165↓^**^	118↑^*^ 309↓^*^ 700↓^*^	n.a.
	Post-	0–20	90↑^***^	X	n.a.	135↓^**^
		20–40	165↑^**^	X	n.a.	80↓^*^ 118↑^*^ 165↑^***^
Control	Pre-	0–20		n.a.	X	90↓^*^
		20–40	118↓^*^ 309↑^*^ 700↑^*^	n.a.	X	
	Post-	0–20	n.a.	135↑^**^	90↑^*^	X
		20–40	n.a.	80↑^*^ 118↓^*^ 165↓^***^		X

*Arrows indicate higher or lower relative abundances of TRFs when conditions are compared from left to right*.

* P < 0.10;

** P < 0.05;

*** *P < 0.01*.

*Empty fields indicate that no differentially expressed TRFs were detected in that comparison. n.a., not analyzed*.

### Effect of experimental flooding on denitrification potentials

**Figure 4:**
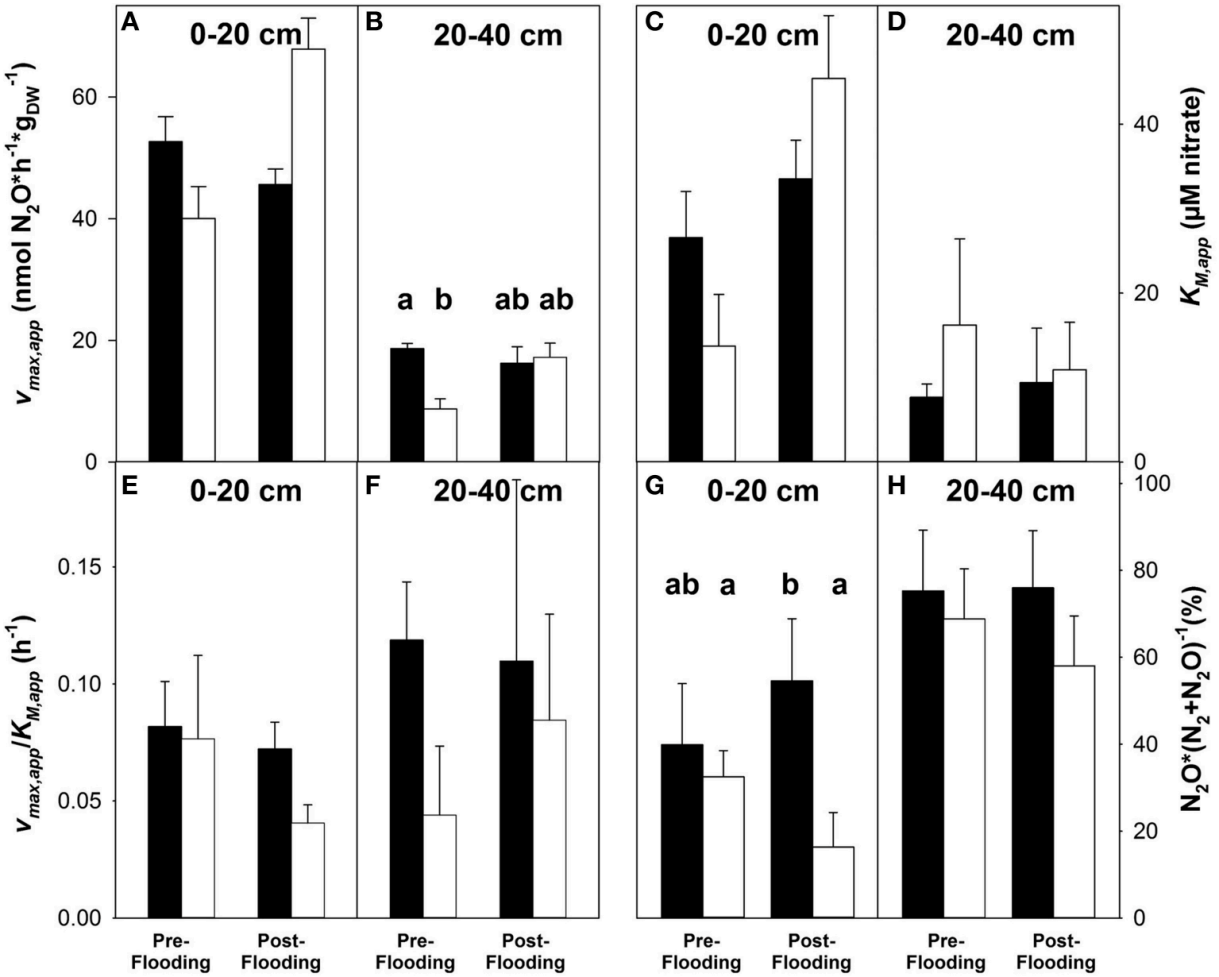
**Effect of prolonged flooding on denitrification potentials in fen soil**. Effect on apparent maximal reaction velocities (*v*_*max, app*_) **(A,B)**, apparent Michaelis-Menten constants (*K*_*M, app*_) **(C,D)**, *v*_*max, app*_*/K*_*M, app*_ **(E,F)**, and N_2_O to (N_2_O + N_2_) ratios **(G,H)** in 0–20 and 20–40 cm fen soil. Values are obtained from apparent Michaelis-Menten kinetics based on mean values and standard errors of 3 replicate measurements (see Figure S5). Black bars represent control plots, white bars represent treatment plots. Siginificantly differing values are indicated by different lower-case letters. Pre-flooding samples were taken on May 11th 2009, post-flooding samples were taken on November 16th 2009.

Fen soil from both soil layers sampled before and after flooding of treatment plots from control and treatment plots produced N_2_O in anoxic microcosms. N_2_O production was minimal in all unsupplemented microcosms (Figure S4). N_2_O production was stimulated without apparent delay in microcosms supplemented with up to 100 μM nitrate (Figure S4). Observed N_2_O prodcution was always higher in the presence than in the absence of acetylene, indicating that part of the N_2_O was further reduced to N_2_ in the absence of acetylene. Initial nitrate-dependent N_2_O production rates of fen soil microcosms amended with acetylene followed apparent Michaelis-Menten kinetics with soil from all soil layers and sampling time points (Figure S5). Apparent maximal reaction velocities (*v*_*max, app*_) ranged from 7 to 68 nmol h^−1^g${}_{\text{dw}}^{-1}$ and were generally higher in 0–20 cm than in 20–40 cm soil (*P* ≤ 0.05; Figures 4A,B). Apparent Michaelis-Menten constants *K*_*M, app*_ ranged from 8 to 45 μM nitrate, but there was no statistically significant difference between the two soil layers (*P* ≥ 0.1; Figures 4C,D). *v*_*max, app*_ was significantly higher in treatment (i.e., flooded) plots after than before flooding in 20–40 cm soil (*p* < 0.001), while it was similar at both time points in control plots and in 0–20 cm soil from treatment plots (*p* ≥ 0.18; Figures 4A,B). *K*_*M*_ was significantly higher after than before flooding in 0–20 cm soil from treatment plots, (*p* = 0.08), while there were no significant differences between *K*_*M*_ in 0–20 cm soil from control plots and in 20–40 cm soil from control and treatment plot at different sampling time points (*p* ≥ 0.2; Figures 4C,D).

The ratio of N_2_O to (N_2_O + N_2_) ranged from 1.9 to 79% and from 38 to 99% for all supplied nitrate concentrations in microcosms with 0–20 and 20–40 cm fen soil, respectively. The average ratio of N_2_O to (N_2_O + N_2_) was 37 and 70% in 0–20 and 20–40 cm fen soil, indicating that more than half of the N_2_O produced from nitrate was further reduced to N_2_ in the upper soil layer (Figures 4G,H). N_2_O to (N_2_O + N_2_) ratios were similar in control plots before and after flooding of treatment plots (*p* ≥ 0.13). This was observed for both sampled soil layers. On the contrary, N_2_O to (N_2_O + N_2_) ratios were significantly lower in 0–20 cm fen soil after than before flooding from treatment plots or than in the samples taken after flooding of treatment plots from the control plots (*p* = 0.01 and *p* < 0.001, respectively). Thus, the results of the microcosm experiments indicate that prolonged flooding enhanced capacities for denitrification as well as N_2_O production concomittant to N_2_O reduction to N_2_.

## Discussion

### Impacts of extreme weather events/short-term water table fluctuations on fen processes and N_2_O source and sink strength

The present study extends existing data on the effects of water table manipulations obtained from peatlands by providing insights into process-associated microbial communities (Martikainen et al., 1993; Silvola et al., 1996; Reiche et al., 2009; Goldberg et al., 2010; Maljanen et al., 2010; Elberling et al., 2011; Estop-Aragonés et al., 2013). Incubation studies indicated higher denitrification potentials but reduced N_2_O to (N_2_O + N_2_) ratios after prolonged flooding (Figure 4). *In situ* N_2_O fluxes from flooded and from control plots differed marginally (Figure 1). Thus, the data indicates that the stimulation of denitrification-derived N_2_O production was essentially mitigated by improved N_2_O reduction capacities. In a recent ^15^N-tracer study, lower N_2_O to (N_2_O + N_2_) ratios were reported under constantly flooded conditions than under fluctuating water tables in a transition bog, supporting the view that complete denitrification is stimulated in flooded peatlands (Tauchnitz et al., 2015). Similar findings were obtained with fresh water marsh, where N_2_O emissions are minimal when the water table is above the peat surface (Yang et al., 2013). Thus, the depletion of nitrate after prolonged flooding coupled with low N_2_O to (N_2_O + N_2_) ratios might prevent higher N_2_O emissions from fen soil, while maintaining its capacity for nitrogen removal.

In Schlöppnerbrunnen fen, N_2_O emissions increased upon rewetting after moderate water table drawdown in 2007 (Goldberg et al., 2010). This was consistent with the literature indicating that rewetting peat sites coincides with high nitrate turnover (Russow et al., 2013). However, N_2_O emissions were rather stable during the more severe water table drawdown in 2008 (Figure 1). During severe water table drawdown, alternative electron acceptors can accumulate (Reiche et al., 2009; Estop-Aragonés et al., 2013) and accumulated nitrate is expected to stimulate denitrification derived N_2_O emissions upon rewetting. However, nitrate accumulation in drought plots was not dramatically higher than in control plots (Estop-Aragonés et al., 2013). Most denitrifiers are heterotrophs depending on organic electron donors (Shapleigh, 2013). Dissolved organic carbon concentrations strongly decreased in response to strong water table drawdown, suggesting that limitations of easily available electron donors did not allow for a stimulation of denitrification and associated N_2_O production upon rewetting relative to control plots (Estop-Aragonés et al., 2013).

### Regulators of denitrification and N_2_O turnover during short-term water table fluctuations

*In situ* denitrifier activity is dependent on a variety of environmental factors such as soil temperature, water table height, and availability of N-oxides. Higher soil temperatures as well as higher soil moisture content generally promote denitrification (Stres et al., 2008; Palmer et al., 2010). Thus, highest sink functions of wetlands for nitrate and N_2_O are observed in summer (Jørgensen and Elberling, 2012). Oxygen availability in peat is mainly controlled by watertable height (Estop-Aragonés et al., 2012). Elevated dissolved oxygen concentrations in the pore water are well known to suppress synthesis of denitrification associated reductases (Tiedje et al., 1982; Shapleigh, 2013). Redox potential changes rather than water content itself impact N_2_O emissions (Liu et al., 2012). However, activities of nitrate, nitrite, nitric oxide, and nitrous oxide reductases display different sensitivities toward oxygen inhibition in model organsims such as *Paracoccus denitrificans* and *Pseudomonas fluorescens* (Davies et al., 1989; McKenney et al., 1994). Nitrate reduction is generally the least oxygen sensitive step. Nitrite, NO, and N_2_O reductions are each increasingly sensitive to oxygen inhibition. Indeed, only the N_2_O reductase is directly inhibited by oxygen (Zumft, 1997). Thus, increased oxygen concentrations due to lowered water tables explain initial increases in N_2_O production observed after moderate water table drawdown in Schlöppnerbrunnen fen and suggest a contribution of denitrifiers to increased N_2_O emissions due to impaired N_2_O reduction (Goldberg et al., 2010). δ^15^N and δ^18^O values of N_2_O suggest a minor contribution, if any, by nitrification (Goldberg et al., 2010). The observed N_2_O consumption of Schlöppnerbrunnen fen when water tables were lowered either naturally in control or experimentally in treatment plots (Figure 1) remains a phenomenon to date that necessitates more research in the future.

Heightened and lowered water tables decrease and increase peat temperature, respectively. In Schlöppnerbrunnen fen, maximal temperatures were 1.5°C lower in flooded than in control plots and about 1°C higher in drought than in control plots (Estop-Aragonés et al., 2012). Given the daily temperature amplitude of 17°C in control plots in 5 cm of depth, such differences appear to be minimal (Estop-Aragonés et al., 2012). Those moderate changes in temperature have the potential to affect denitrifier activities, but do not necessarily change denitrifier community composition (Stres et al., 2008). Indeed, denitrifier community composition in Schlöppnerbrunnen fen remained similar at all time points of the manipulation experiments (Figure 3; Figures S2, S3). During periods of drought, enhanced rates of nitrification are feasible due to elevated oxygen availability (Fromin et al., 2010). In Schlöppnerbrunnen fen soil, nitrate concentrations in the pore water increased upon moderate water level draw down up to 500 μM and decreased after rewetting (Herrmann et al., 2012). Although nitrate was not significantly increased due to strong water level drawdown, concentrations of other terminal electron acceptors such as Fe^3+^ and sulfate increased during drying and decreased during rewetting, suggesting a buffering capacity for high redox potentials in the fen (Estop-Aragonés et al., 2013). Such a buffering capacity together with carbon limitation might have prevented a major stimulation of denitrification after rewetting.

During flooding, constant inputs of nitrate and sulfate raised their concentrations in the peat to ~40 and 100 μM, respectively (Estop-Aragonés et al., 2013). The constant supply of nitrate in low concentrations might lead to growth and activation of fen denitrifiers, which are often N-limited, and increased nitrate supply might lead to increased N_2_O emissions (Novak et al., 2015; Palmer and Horn, 2015). Along these lines, denitrification capacities of Schlöppnerbrunnen fen were higher after than before flooding in treatment plots (Figures 4A–F). Potential N_2_O to (N_2_O + N_2_) ratios (Figures 4G,H) tended to be lower after than before flooding, while the abundance of denitrification associated genes remained rather unaffected or tended to decrease (Figure 2). Model denitrifiers such as *P. denitrificans* and *P. fluorescens* are capable of minimizing N_2_O-release during complete denitrification under stable anoxic conditions by a stable expression of denitrification associated reductases (McKenney et al., 1994; Baumann et al., 1996). Such data suggest that the nitrate input during flooding did not allow for massive growth of denitrifiers, and that the prevalent denitrifier community is regulated in a way that the conversion of N_2_O to N_2_ *in situ* was efficient.

Water table manipulation studies in wetland soils indicate that the effect of short-term water table fluctuations on denitrifier abundance is variable, ranging from no effect to decreased or increased abundances (Kim et al., 2008; Song et al., 2010). Differences in denitrifier activity are observed after short-term water table manipulations in many wetland systems (Kim et al., 2008; Song et al., 2010). In Schlöppnerbrunnen fen, the relative abundance of detected *narG* was rather unaffected by the drought and flooding treatments (Figures 2E–H), while the relative abundance of detected *nosZ* was higher after drought in upper fen soil (Figure 2I). Song et al. (2010) concluded that short-term water table variations impact denitrifier activity rather than denitrifier community structure. Similar effects have been observed for methanogenic communities: While increased substrate availability increases methanogenic activity, the community composition of methanogens is rather unaffected by anthropogenic disturbances (Basiliko et al., 2013). Thus, the observed changes in denitrification potentials and N_2_O emission in Schlöppnerbrunnen fen appear to be caused by changes in denitrifier activity rather than by changes in denitrifier community size.

### Fen denitrifiers are resistant to climate change induced short-term water table fluctuations and are capable to adapt their activity to changing redox conditions

Denitrifier community composition is rather unaffected by water table fluctuations in many soils (Stres et al., 2008; Song et al., 2010). Certain microbial communities are resistant to environmental stress, such as water table fluctuations, varying temperatures or freeze-thaw events (Griffiths and Philippot, 2013). Denitrifier communities are also rather stable to water table fluctuations in other wetland soils such as in Ohio wetlands, saltmarshes or wetland ponds (Fromin et al., 2010; Song et al., 2010; McKew et al., 2011). Indeed, *narG* and *nosZ* copy numbers were only marginally affected by the water table manipulations in Schlöppnerbrunnen fen (Figures 2E–L), indicating that facultative fen denitrifiers are able to cope with changing water tables and the resulting changes in oxygen supply. Moreover, DNA-based TRFLP analyses indicate a stable denitrifier community composition (Figure 3), i.e., resistance to water table changes is similar in most groups of fen denitrifiers. Earlier studies with Schlöppnerbrunnen fen soil indicate the presence of nitrate reducers including denitrifiers related to *Deinococcus*-*Thermus, Actinobacteria* as well as *Alpha-* and *Beta*-*Proteobacteria* (Palmer et al., 2010). Also the present study detected TRFs indicating the presence of such groups on both gene and transcript level (Figures S2, S3). Thus, based on transcript level TRFLP analysis, *Deinococcus*-*Thermus* related microbes, *Proteobacteria* as well as *Actinobacteria* were active in Schlöppnerbrunnen fen under variable environmental conditions. *Deinococcus* -*Thermus* related microbes represent a deep-branching group that are widespread in extreme environments and resistant to environmental stress (da Costa et al., 2006; Theodorakopoulos et al., 2013). *Proteobacteria* are found in most soil ecosystems and under a variety of environmental conditions due to their versatile metabolic capabilities (Dworkin et al., 2006). *Actinobacteria* are likewise common to many soils, frequently occur in more extreme habitats, and show high tolerance to environmental stress (Zenova et al., 2011). Many *Actinobacteria* possess a truncated denitrification pathway, and NO or N_2_O are often end products of *Actinobacterial* denitrification (Shapleigh, 2013). Schlöppnerbrunnen fen emits up to 1 μmol NO m^−2^ h^−1^, demonstrating significant production and stability of NO to act as biological signal molecule (Goldberg et al., 2010). Less than 1 nM concentrations of NO suffice to induce *norBC* expression and 5 nM of NO result in maximal expression of *norBC* as well as *nirS* in *Pseudomonas stutzeri* (Vollack and Zumft, 2001). Thus, the NO produced in peatlands (eventually by incomplete denitrifiers like *Actinobacteria*) might act as an activator for the denitrifying microbial community in peatland soils by inducing the expression of denitrification genes (i.e., those of detected *Proteobacteria*).

The versatility of such peatland denitrifiers likely contributed to the observed stability of the denitrifier community. The stability of the Schlöppnerbrunnen fen denitrifier community was corroborated by the absence of significant seasonal variations in control plots (Figure 3). Boreal lake sediments likewise host a rather stable denitrifier community throughout most sampling times during a year (Saarenheimo et al., 2015). However, such findings are in contrast to other studies of agricultural soils, drained peatlands and intertidal wetland ecosystems where season significantly impacted denitrifier community composition (Bremer et al., 2007; Marhan et al., 2011; Andert et al., 2012; Hu et al., 2014; Wang et al., 2014). During such studies a developing plant community, seasonally changes of environmental parameters such as pH, and sampling times that covered the whole year including winter contributed to the observed seasonal changes in denitrifier community. During our study, sampling was restricted to the time periods from June until August (experimental drought) and from May to November (experimental flooding) and established plant communities were rather stable. Minor community changes might have escaped detection by TRFLP analysis and few species sensitive to water table manipulations might have been replaced by others yielding a similar TRF. Other factors such as nutrient availability might affect the resistance of the microbial community (Royer-Tardif et al., 2010; Liu et al., 2012). Fluctuating water tables and thus redox conditions occur frequently in Schlöppnerbrunnen fen soil, thus an adaptation and hence stability of microbial communities toward redox fluctuations and changing environmental conditions is likely. Indeed, laboratory studies on soil microbes lend support for such a conclusion (Pett-Ridge and Firestone, 2005).

Although denitrifier community structure was stable and the effect of water table manipulations on *in situ* N_2_O fluxes was low, water table manipulations affected potential activities and active denitrifiers (Figures 2, 3). Microcosm experiments with Schlöppnerbrunnen fen soil indicate increased *v*_*max*_ after flooding as well as decreased N_2_O to (N_2_O + N_2_) ratios (Figure 4), and cDNA-based TRFLP analyses of *narG* and *nosZ* indicate differences in the active denitrifier community at different water table regimes (Figure 3). Short-term water table fluctuations affect denitrifier activity (Fromin et al., 2010; Song et al., 2010). Denitrifier activities do not always correlate with denitrifier community structure (Andert et al., 2012). Thus, the observed stability of the fen denitrifier community composition during the vegetation period and short-term water table fluctuations might be an interesting feature that might be more common than previously thought. Observed differences in N_2_O emission are likely caused by changes in substrate availability and denitrifier activity rather than by changes in community composition.

## Conclusions and limitations

Denitrifier communities are diverse, the denitrification pathway is modular, and the knowledge on existing forms of N-oxide respiring enzymes is growing constantly. Recently, atypical *nosZ* belonging to the clade II have been described that occur in microbes lacking modules of the denitrification pathway and those atypical *nosZ* can account for more than half of the *nosZ* in soil (Jones et al., 2013; Orellana et al., 2014). Organisms hosting clade II *nosZ* can be denitrifiers or non-denitrifiers. Many non-denitrifying N_2_O-reducers are obligate anaerobes rather than facultative aerobes, suggesting that they have a higher sensitivity toward redox fluctuations than denitrifiers. However, their importance in peatlands is unclear to date, and thus their role has to be further clarified in future studies. The present study focused on denitrifers, and the molecular analyses were conducted with primers targeting clade I *nosZ* of denitrifiers. Due to selectivities of primers, microbial abundance and diversity might be underestimated. However, even though the present study captures only part of the genetic denitrifier diversity, trends observed for detected genes and transcripts are valid, and the collective data of the study indicate (i) rather stable *in situ* N_2_O fluxes during drought and flooding experiments, (ii) increased potential activity of fen denitrifiers as well as a higher percentage of complete denitrification after prolonged flooding, (iii) a stable denitrifier community in Schlöppnerbrunnen fen soil that is resistant to short-term water table fluctuations, (iv) a potential of the core denitrifier community to react to fluctuating water tables by differential activation, and (v) the ability of fen denitrifiers and eventually non-denitrifying N_2_O reducers to consume N_2_O under moderately acidic conditions. Those findings support the hypothesis that short-term water table fluctuations affect denitrifier activity rather than their community composition. It is feasible that enhanced overall denitrification rates as they can be expected under certain conditions (e.g., after prolonged flooding) and enhanced N_2_O consumption rates equal out, thus leading to rather stable overall N_2_O fluxes.

## Author contributions

Conceived and designed the experiments: KP, JK, GG, and MH. Performed the experiments: KP and JK. Analyzed the data: KP, JK, GG, and MH. Contributed reagents/materials/analysis tools: MH and GG. Wrote the paper: KP and MH.

### Conflict of interest statement

The authors declare that the research was conducted in the absence of any commercial or financial relationships that could be construed as a potential conflict of interest.

## Supplementary material

Figure S1 **Effect of artificial drought (2008) and prolonged flooding (2009) on watertables in control and treatment plots**. Negative values indicate a watertable below the peat surface, positive values indicate a watertable above the peat surface. Averages of 18 piezometer measurements (6 per plot) are displayed. Error bars have been omitted to improve picture clarity. Sampling time points are indicated by vertical black lines.

Figure S2 **Effect of experimental drought (A–D) and prolonged flooding (E–H) on community composition of *narG* in DNA (A,B,E,F) and cDNA (C,D,G,H) in fen soil from 0 to 20 cm (A,C,E,G) and 20 to 40 cm (B,D,F,H) depth**. PCR products were digested with *Cfo*I. Mean values of values from all plots of one plot type and soil depths 0–20 or 20–40 cm (i.e., 3 × 2 = 6) are displayed. TRFs with a relative abundance < 5% in all samples are combined as “Rare.” Pre-drought samples were taken on June 09th 2008, post-drought samples were taken on July 27th 2008. Pre-flooding samples were taken on May 11th 2009, post-flooding samples were taken on November 16th 2009. TRFLP patterns were dominated by TRFs of 23, 57, and 128 bp size, which were indicative of FEN CLUSTER 7 (*Deinococcus-Thermus* sp.-related uncultured soil bacteria), FEN CLUSTER 6 (*Actinobacteria*-related uncultured soil bacteria), and FEN CLUSTERS 1–5 (related to uncultured Proteobacteria).

Figure S3 **Effect of experimental drought (A–D) and prolonged flooding (E–H) on community composition of *nosZ* in DNA (A,B,E,F) and cDNA (C,D,G,H) in fen soil from 0 to 20 cm (A,C,E,G) and 20 to 40 cm (B,D,F,H) depth**. PCR products were digested with *Fnu4*HI. Mean values of values from all plots of one plot type and soil depths 0–20 or 20–40 cm (i.e., 3 × 2 = 6) are displayed. TRFs with a relative abundance < 5% in all samples are combined as “Rare.” Pre-drought samples were taken on June 09th 2008, post-drought samples were taken on July 27th 2008. Pre-flooding samples were taken on May 11th 2009, post-flooding samples were taken on November 16th 2009. In DNA samples, the five most prominent TRFs (149, 165, 215, 298, 700 bp) had cumulative relative abundances of 82–99% in all treaments and sampling time points in 2008 and 2009. In cDNA samples, those same TRFs accounted only for 22–52% and 38–90% of the TRFs in 2008 and 2009, respectively. The TRFs were indicative of FEN CLUSTER 1 (TRFs 165, 298; affiliated with *Bradyrhizobiaceae-*related uncultured soil bacteria) and FEN CLUSTERS 3–5 (TRFs 149, 215; affiliated with *Rhodospirillaceae*-related uncultured soil bacteria).

Figure S4 **Effect of acetylene and supplemental nitrate on the production and consumption of N_2_O in anoxic microcosms with 0–20 cm (1) and 20–40 cm (2) fen soil**. Blue squares represent the pre-flooding control plot, red circles represent the pre-flooding treatment plot, black triangles represent the post-flooding control plot, green diamonds represent the post-flooding treatment plot. Closed symbols represent microcosms with acetylene, open symbols represent microcosms without acetylene. Supplied concentrations of nitrate were 0 μM **(A)**, 10 μM **(B)**, 20 μM **(C)**, 40 μM **(D)**, and 100 μM **(E)**. Mean values and standard errors of three replicate microcosms are shown.

Figure S5 **Effect of prolonged flooding on apparent Michaelis-Menten kinetics in peat soil from 0 to 20 cm (A) and 20 to 40 cm (B) peat soil**. N_2_O-production rates were measured at different nitrate concentrations in microcosms that were supplemented with acetylene. Averages of three measurements and standard errors are displayed. Open symbols = control plots, closed symbols = treatment plots. Squares = pre-flooding, circles = post-flooding.
