# Supplementary figures and images for: Drying-Rewetting and Flooding Impact Denitrifier Activity Rather than Community Structure in a Moderately Acidic Fen
Source: Front Microbiol. 2016 Jun 1;7:727. doi: 10.3389/fmicb.2016.00727 (PMC4887476; doi:10.3389/fmicb.2016.00727)

## Slide 1
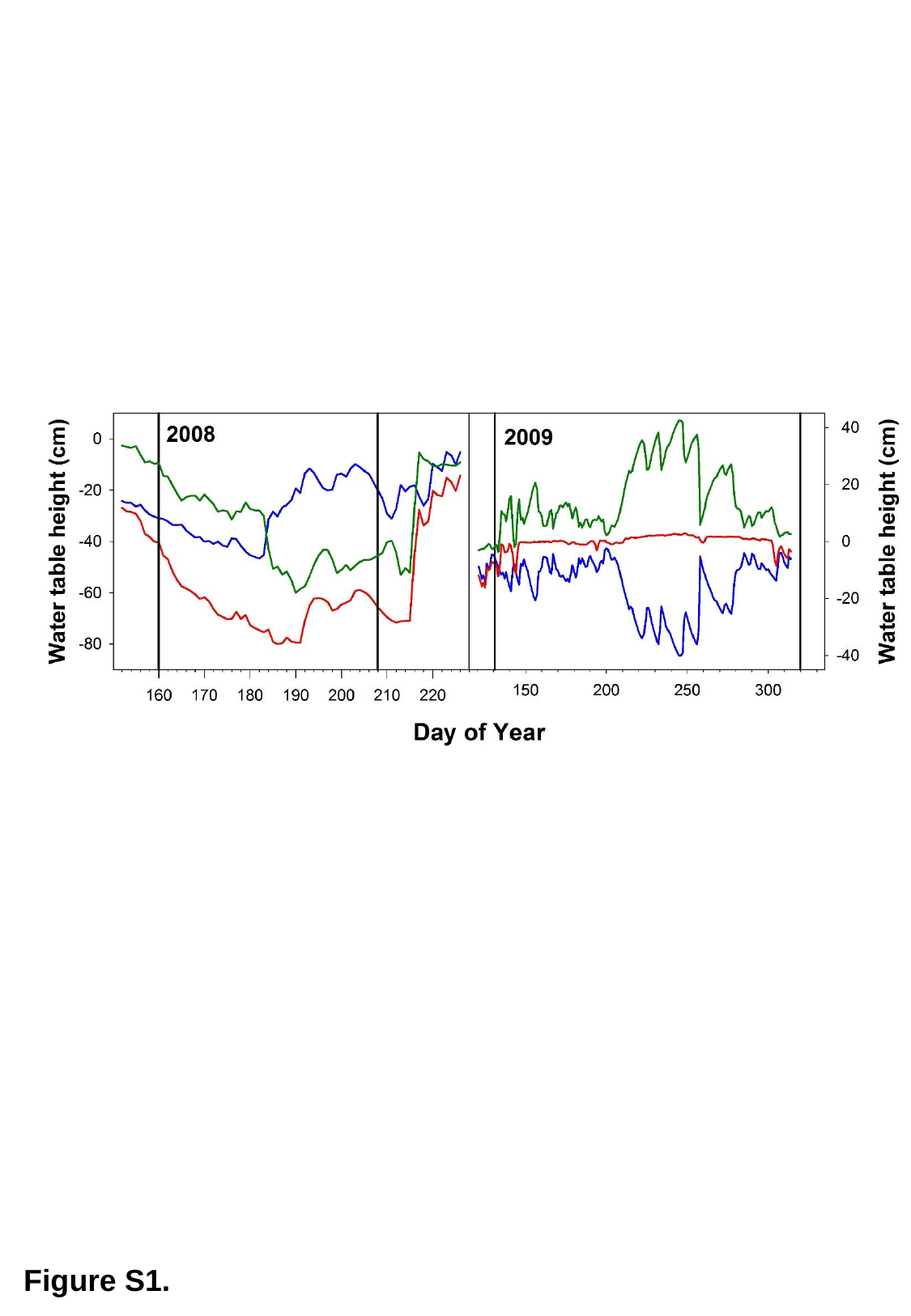

Figure S1.

## Slide 2
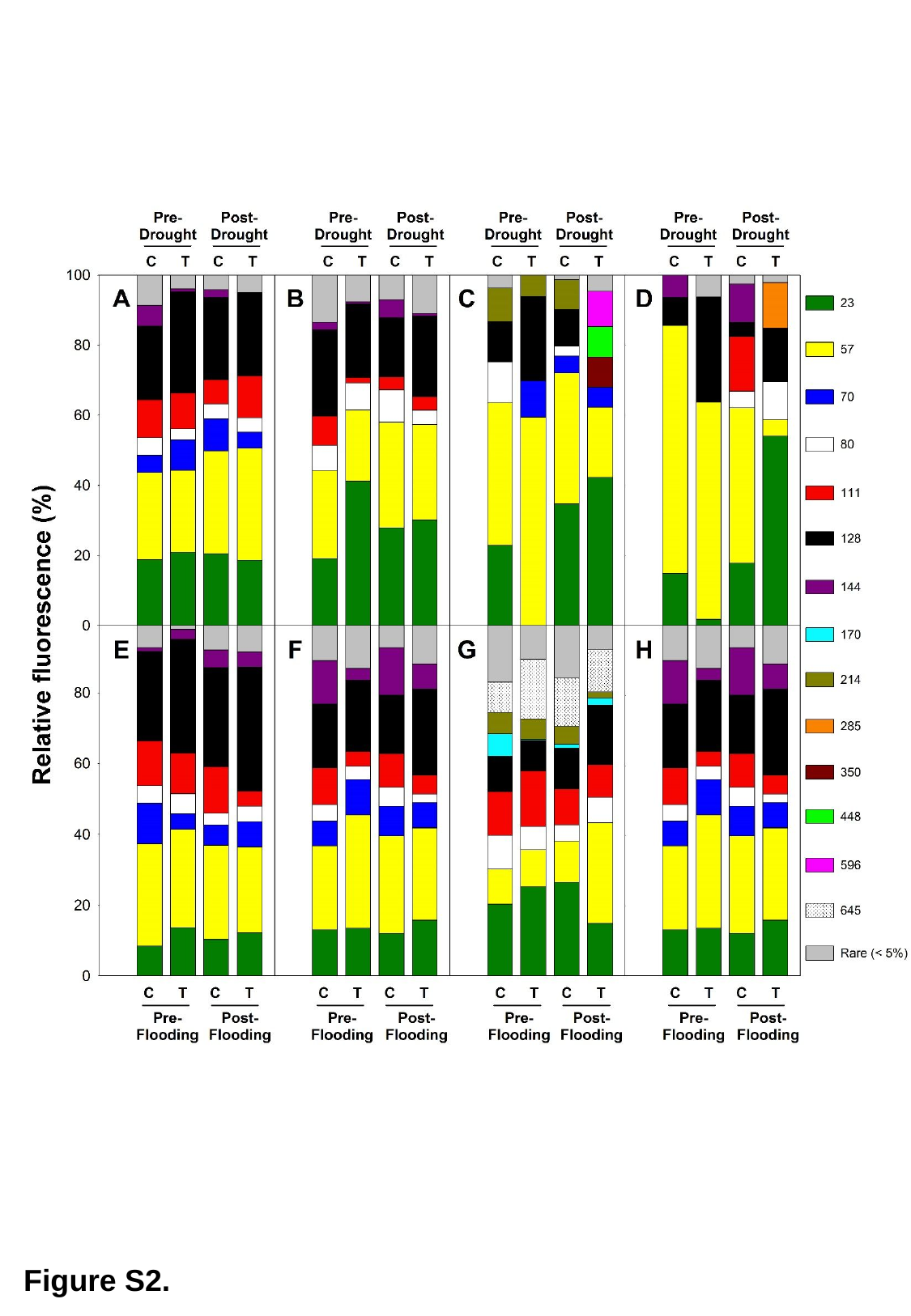

Figure S2.

## Slide 3
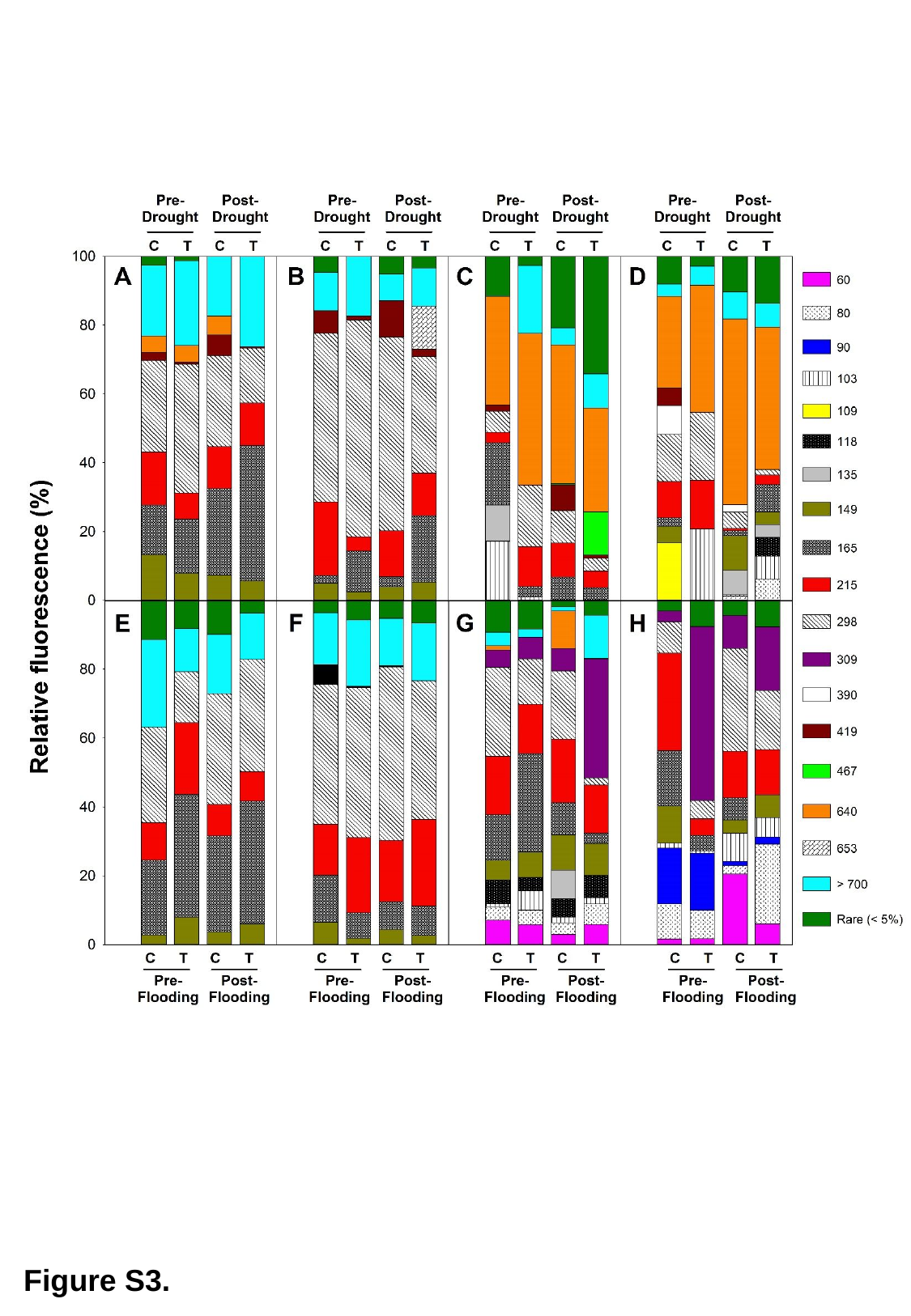

Figure S3.

## Slide 4
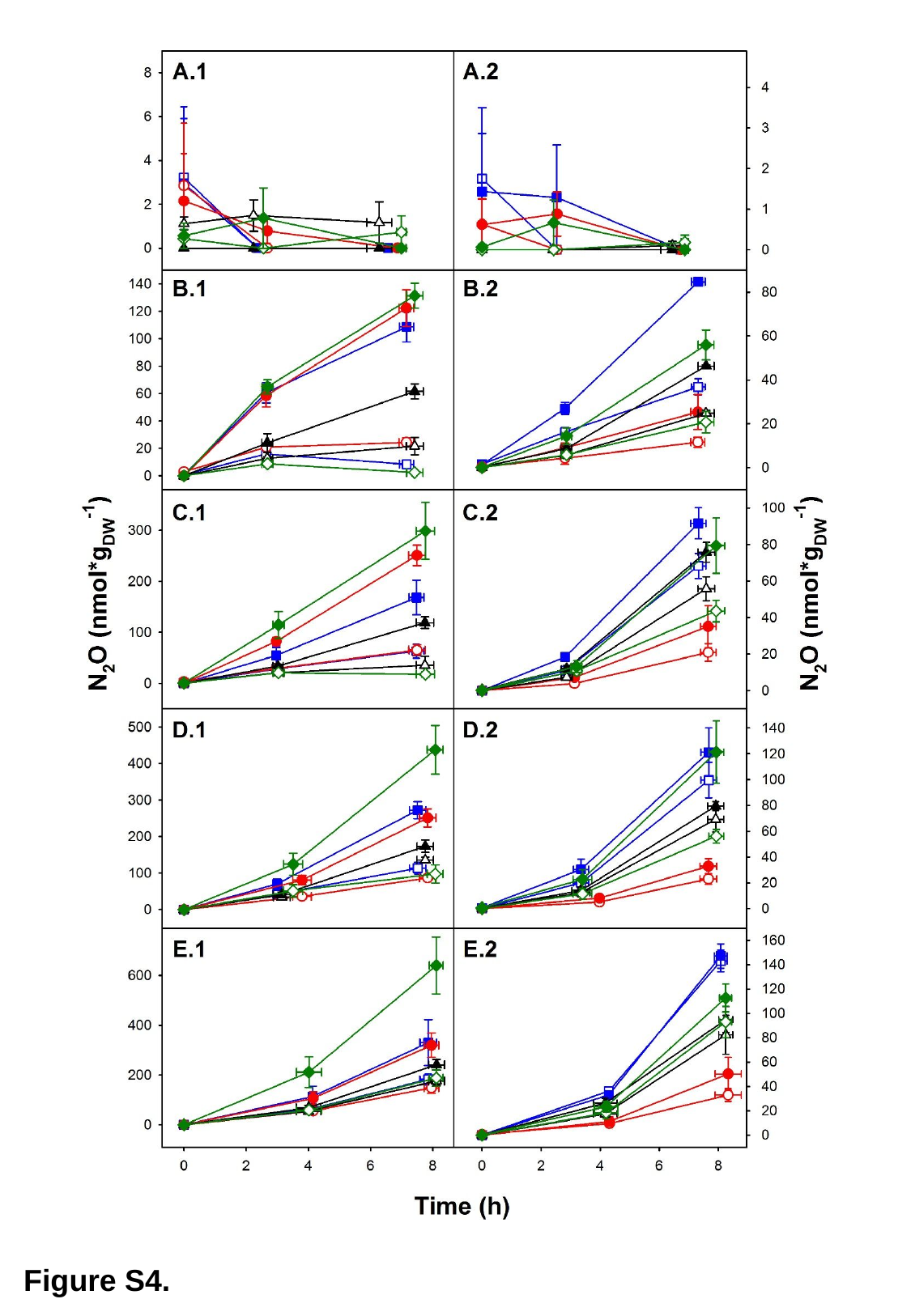

Figure S4.

## Slide 5
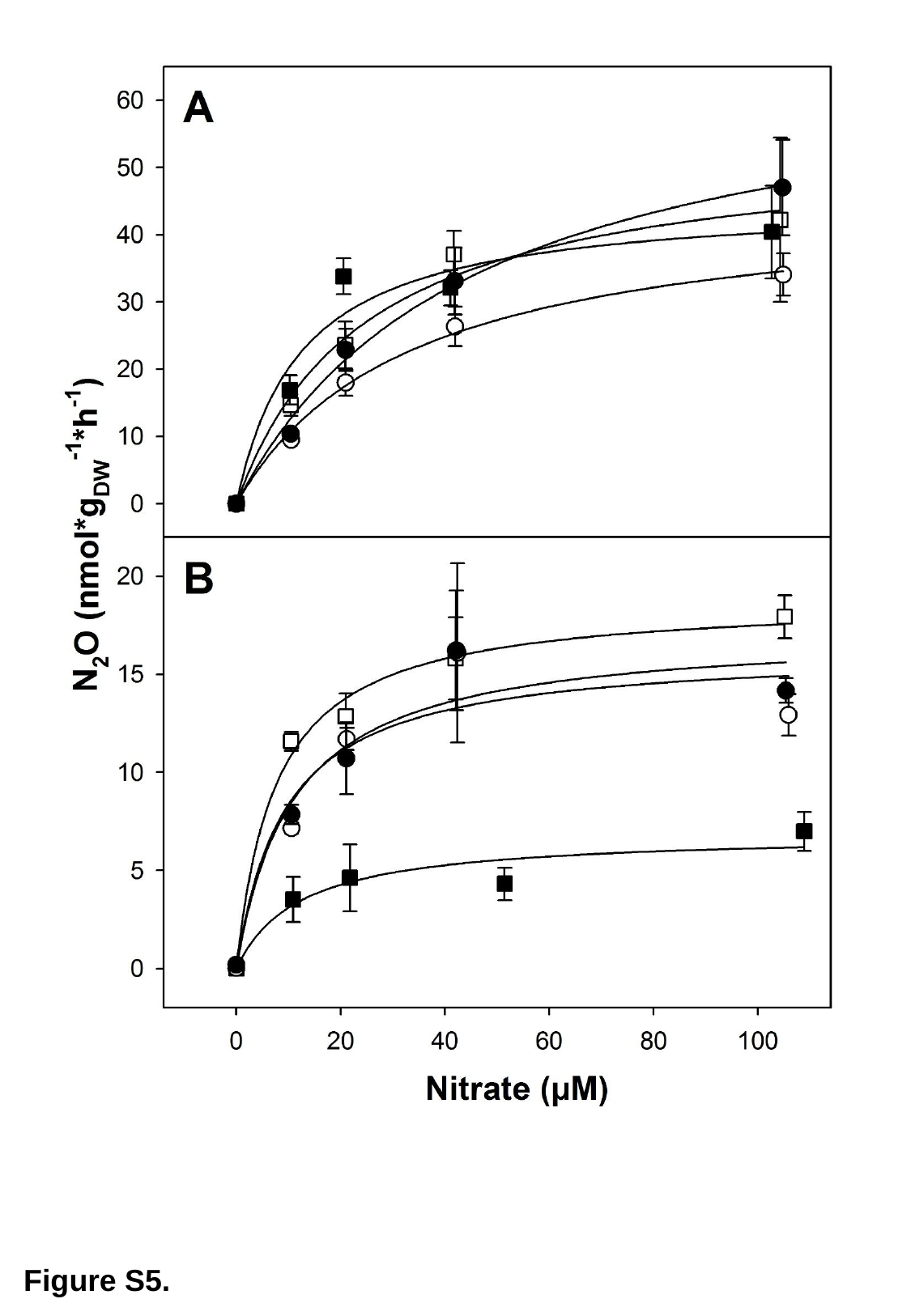

Figure S5.

Supplement: Figure S1 — Effect of artificial drought (2008) and prolonged flooding (2009) on watertables in control and treatment plots. Negative values indicate a watertable below the peat surface, positive values indicate a watertable above the peat surface. Averages of 18 piezometer measurements (6 per plot) are displayed. Error bars have been omitted to improve picture clarity. Sampling time points are indicated by vertical black lines. [file Presentation1.PPTX]
